# TSCQ study: a randomized, controlled, open-label trial of daily trimethoprim-sulfamethoxazole or weekly chloroquine among adults on antiretroviral therapy in Malawi: study protocol for a randomized controlled trial

**DOI:** 10.1186/s13063-016-1392-3

**Published:** 2016-07-18

**Authors:** Matthew B. Laurens, Randy G. Mungwira, Osward M. Nyirenda, Titus H. Divala, Maxwell Kanjala, Francis Muwalo, Felix A. Mkandawire, Lufina Tsirizani, Wongani Nyangulu, Edson Mwinjiwa, Terrie E. Taylor, Jane Mallewa, William C. Blackwelder, Christopher V. Plowe, Miriam K. Laufer, Joep J. van Oosterhout

**Affiliations:** Division of Malaria Research, Institute for Global Health, University of Maryland School of Medicine, 480 W Baltimore St, Room 480, Baltimore, MD 21218 USA; Blantyre Malaria Project, Blantyre, Malawi; Dignitas International, Zomba, Malawi; College of Osteopathic Medicine, Michigan State University, East Lansing, MI USA; University of Malawi College of Medicine, Blantyre, Malawi; Institute for Global Health, University of Maryland School of Medicine, Baltimore, MD USA

**Keywords:** HIV infection, Trimethoprim-sulfamethoxazole, Chloroquine, Immunosuppression, Longitudinal studies, Malaria, *Plasmodium falciparum*, Parasitemia, Adult, Antiretroviral therapy, Malawi, Africa

## Abstract

**Background:**

Before antiretroviral therapy (ART) became widely available in sub-Saharan Africa, several studies demonstrated that daily trimethoprim-sulfamethoxazole (TS) prophylaxis reduced morbidity and mortality among HIV-infected adults. As a result, the World Health Organization (WHO) recommended administering TS prophylaxis to this group. However, the applicability of the results to individuals taking ART and living in sub-Saharan Africa has not been definitively evaluated. This study aims to determine if TS prophylaxis benefits HIV-infected Malawian adults after a good response to ART. If TS prophylaxis does indeed show benefit, it is important to determine if this is due to its antibacterial and/or antimalarial properties.

**Methods/design:**

A randomized, controlled, open-label, phase III trial of continued standard of care prophylaxis with daily trimethoprim-sulfamethoxazole (TS) compared to discontinuation of standard of care TS prophylaxis and starting weekly chloroquine (CQ) prophylaxis or discontinuation of standard of care TS prophylaxis. The study will randomize 1400–1500 HIV-infected adults (equally divided over the three study arms) with a nondetectable viral load and a CD4 count of 250/mm^3^ or more from antiretroviral therapy clinics in Blantyre and Zomba. The expected rate of primary endpoint events of death and WHO stage 3 and 4 events is 6.8 per 100 person-years of follow-up in all participants. Assuming the number of events follows a Poisson distribution and average participant follow-up after 10 % loss to follow-up is 41.6 months, the study will have approximately 85 % power to rule out a reduction of 35 % or more in primary endpoint events in the TS or CQ arms compared to discontinuation of TS prophylaxis—i.e., to show that discontinuation of TS prophylaxis is noninferior to either TS or CQ, with a noninferiority margin of 35 %. Ethical and regulatory approvals were obtained from the University of Malawi College of Medicine Research Ethics Committee; the Malawi Pharmacy, Medicines and Poisons Board; and the University of Maryland Baltimore Institutional Review Board.

**Discussion:**

The study began recruitment activities at the Ndirande site in November 2012. The sponsor agreed to extend and expand the study in early 2015, and a second site, Zomba, was added for recruitment and follow-up in mid-2015.

**Trial registration:**

ClinicalTrials.gov Identifier: NCT01650558 (registered on 6 July 2012).

**Protocol version:**

Letter of amendment #1 to the DAIDS-ES 10822 TSCQ Malawi Protocol, Version 4.0, 16 December 2014.

**Electronic supplementary material:**

The online version of this article (doi:10.1186/s13063-016-1392-3) contains supplementary material, which is available to authorized users.

## Background and rationale

### Background

Several studies have demonstrated that daily trimethoprim-sulfamethoxazole (TS) prophylaxis reduces morbidity and mortality among HIV-infected adults in sub-Saharan Africa [1–4]. As a result of these studies the World Health Organization (WHO) recommended administering TS prophylaxis to this group. However, these studies were completed prior to the widespread availability of antiretroviral therapy (ART), and the applicability of the results to individuals taking ART has not been definitively evaluated. A critical question remains about the need for, and duration of, TS prophylaxis and its public health impact: Is there a benefit to TS prophylaxis after patients have initiated and responded to ART?

In North America and Europe, TS prophylaxis prevents *Pneumocystis jirovecii* pneumonia (PCP) and toxoplasmosis, and is discontinued after the CD4 cell count reaches 200/mm^3^. However, a recent multicenter study demonstrated that even with a CD4 cell count of 100–200/mm^3^, there is minimal benefit of TS prophylaxis if the patient is taking ART and the viral load is undetectable [5]. The risk of opportunistic infections, at least those infections common in Western countries, is very low once ART is successful, even with low CD4 cell counts.

In contrast, studies in Africa have not determined whether there is a point when TS prophylaxis no longer confers an advantage with respect to survival or morbidity. There is consistent evidence to support the use of a CD4 cell count of more than 200 cells/mm^3^ as a threshold above which TS prophylaxis survival benefit is absent among individuals receiving ART [3, 6]. However, the broad benefit of TS to prevent bacteremia, pneumonia, enteritis and malaria has not been consistently documented in adults taking ART who live in sub-Saharan Africa. In the Development of Antiretroviral Therapy in Africa (DART) study, the only clear disease-specific benefit of TS was in the prevention of malaria; TS did not prevent WHO clinical stage 4 illnesses [6]. A clinical trial to assess the benefit of TS prophylaxis in a population of Ugandan adults taking ART was stopped early because the group taking TS prophylaxis had fewer nonsevere clinical events, such as uncomplicated malaria and diarrheal illnesses, than the control group [7]. A similar study of TS discontinuation was recently conducted in Ugandan adults to assess the TS prophylaxis benefit with respect to hematological adverse events [8]. Although the main outcome is not yet published, investigators did report a decrease in malaria among participants taking TS therapy [9]. Another study randomized Kenyan adults taking ART to continue or stop TS prophylaxis, and found that after 1 year of follow-up, TS did not change morbidity and mortality with the exception of fewer malaria cases in the TS arm, and no changes in CD4 count or ART failure were noted over time [10]. Malaria prevention is potentially important, as it is associated with transient increases in HIV viral load [11]. However, it is not known if these increases are associated with loss of virologic suppression leading to HIV disease progression.

At the study sites, daily TS prophylaxis is currently used by all persons taking ART [12], and is recommended to be continued for life in the absence of severe side effects as there are no randomized, controlled trials to determine whether or when to stop TS in persons taking ART.

### Rationale

The role of TS prophylaxis in the context of long-term ART is an important issue now facing ART programs in Africa. Currently, the updated WHO recommendation calls for TS prophylaxis in those with a CD4 count of 350 cells/mm^3^ or less in areas where bacterial infection and malaria are prevalent [13]. However, this recommendation is based on expert opinion and not on randomized, controlled trials. Some data from resource-limited settings suggest that it may be safe to discontinue TS among those with immune recovery and a CD4 count above 200 cells/mm^3^ in response to ART [14, 15]. To date, no randomized clinical trials have assessed the safety and timing of discontinuation of TS prophylaxis following immune recovery in response to ART in resource-limited settings [16].

The main study hypotheses are that TS confers a long-term benefit on survival and disease control and that the benefit is largely attributable to the prevention of malaria. The study design takes advantage of the unique opportunity in Malawi, where malaria is uniformly susceptible to CQ [17, 18]. In addition to a TS continuation arm and a TS discontinuation control arm, a third arm receives only CQ prophylaxis. Individuals who receive CQ will be receiving extremely effective malaria prevention but will not be protected against bacterial infection. Thus, one study arm prevents both bacterial and parasitic infection, a second arm prevents just malaria and the third arm will not receive prophylaxis.

This study will inform HIV policy throughout Africa. TS prophylaxis at approximately one billion doses/year could potentially be removed from the ART regimen or, if it is beneficial, its use could be more broadly reinforced as a method of saving lives and prolonging the efficacy of the available ART. A control arm that discontinues TS prophylaxis after good clinical and virologic response to ART is included in the study because of clinical equipoise regarding the benefit of prophylaxis in this population. A randomized clinical trial is needed to definitively inform health policy in Malawi and other sub-Saharan African countries that currently continue TS prophylaxis after successful ART therapy [16].

### Overview of the trial

A randomized, controlled, open-label, phase III trial of continued standard of care prophylaxis with daily TS compared to (1) discontinuation of standard of care TS prophylaxis and starting weekly chloroquine (CQ) prophylaxis, or (2) discontinuation of standard of care TS prophylaxis.

Study aim: to assess the need for antimicrobial prophylaxis in HIV-infected adults taking ART in sub-Saharan Africa.

Intervention: continue daily TS, stop daily TS, or stop daily TS and start weekly CQ.

Randomization: 1:1:1 to each study intervention, using block randomization and stratified by site.

Primary endpoint: the occurrence of a severe event (WHO stage 3 or 4 event or death, see Table 1).

**Table 1:** Revised World Health Organization clinical staging for HIV/AIDS for adults and adolescents with confirmed HIV infection

Clinical stage 2
• Moderate unexplained weight loss (5–10 % of presumed or measured body weight)
• Recurrent respiratory tract infections (sinusitis, tonsillitis, otitis media and pharyngitis)
• Herpes zoster
• Angular cheilitis
• Recurrent oral ulceration
• Papular pruritic eruptions
• Seborrhoeic dermatitis
• Fungal nail infections
Clinical stage 3
• Unexplained severe weight loss (>10 % of presumed or measured body weight)
• Unexplained chronic diarrhoea for longer than 1 month
• Unexplained persistent fever (above 37.5 °C intermittent or constant, for longer than 1 month)
• Persistent oral candidiasis
• Oral hairy leukoplakia
• Pulmonary tuberculosis
• Severe bacterial infections (e.g., pneumonia, empyema, pyomyositis, bone or joint infection, bacteremia, severe pelvic inflammatory disease)
• Acute necrotizing ulcerative stomatitis, gingivitis or periodontitis
• Unexplained anemia (<8 g/dl), neutropenia (<500/mm^3^) and/or chronic thrombocytopenia (<50,000/mm^3^)
Clinical stage 4
• HIV wasting syndrome
• *Pneumocystis jirovecii* pneumonia
• Recurrent severe bacterial pneumonia
• Chronic herpes simplex infection (orolabial, genital or anorectal of more than 1 month’s duration or visceral at any site)
• Oesophageal candidiasis (or candidiasis of the trachea, bronchi or lungs)
• Extrapulmonary tuberculosis
• Kaposi’s sarcoma
• Cytomegalovirus infection (retinitis or infection of other organs, excluding liver, spleen or lymph nodes)
• Central nervous system toxoplasmosis
• HIV encephalopathy
• Extrapulmonary cryptococcosis including meningitis
• Disseminated nontuberculous mycobacterial infection
• Progressive multifocal leukoencephalopathy (PML)
• Candida of trachea, bronchi or lungs
• Chronic cryptosporidiosis
• Chronic isosporiasis
• Disseminated mycosis (histoplasmosis, coccidiomycosis)
• Recurrent septicemia (including nontyphoidal *Salmonella*)
• Lymphoma (cerebral or B-cell non-Hodgkin)
• Invasive cervical carcinoma
• Atypical disseminated leishmaniasis
• Symptomatic HIV-associated nephropathy or symptomatic HIV-associated cardiomyopathy

Antiretroviral therapy: continued in all participants as per Malawi Ministry of Health guidelines.

Secondary endpoints: undetectable (less than 400 copies/mL) HIV viral load and CD4 cell count assessed every 24 weeks, incidence of WHO HIV stage 2-, 3- or 4-defining illnesses or death, the occurrence of any suspected and laboratory-confirmed infection with bacteria or malaria, the occurrence of grade 3 or above adverse events (AEs) and rate of discontinuation of TS or CQ prophylaxis.

Sample size: 1400–1500 participants (approximately 1000–1100 from Blantyre and 400–500 from Zomba).

Study sites: Blantyre and Zomba, Malawi.

Study sponsor: US National Institutes of Health, National Institute of Allergy and Infectious Diseases, Division of Acquired Immunodeficiency Syndrome.

Study duration (recruitment and follow-up): 5.5 years.

### Study objectives

#### Primary objective

To determine if prophylaxis with TS or CQ, compared to no prophylaxis, is associated with improved morbidity and mortality among adults receiving ART beyond 6 months.

#### Secondary objectives

The secondary objectives are as follows: (1) to assess the effect of prophylaxis with TS or CQ on the virologic, immunologic and clinical response to ART, (2) to assess the efficacy of TS in preventing infection with bacteria or malaria, and (3) to assess the safety and tolerability of TS and CQ prophylaxis.

#### Exploratory objectives

Exploratory objectives include the following: (1) to evaluate the effect of TS and CQ prophylaxis on the incidence of drug-resistant organisms, and (2) to evaluate the efficacy of antimalarial treatment.

### Primary endpoint

The occurrence of a severe event (WHO stage 3 or 4 event or death).

### Secondary endpoints

#### Virologic: undetectable (less than 400 copies/mL) HIV viral load assessed every 24 weeks

Intercurrent infection may increase HIV viral load replication and could lead to expansion of ART-resistant HIV and loss of viral suppression. We will compare the ability of prophylactic TS and CQ to prevent loss of viral suppression compared to no prophylaxis.

#### Immunologic: CD4 cell count assessed every 24 weeks

During intercurrent illness, the CD4 count may decline and lead to increased susceptibility to opportunistic infections. Prevention of infection with TS or CQ prophylaxis could avoid this decline and worsening of immunosuppression. We will analyze the ability of prophylactic TS and CQ to prevent CD4 cell count decline compared to no prophylaxis.

#### Clinical: incidence of WHO HIV stage 2-, 3- or 4-defining illnesses or death

In addition to the severe event outcomes listed in the primary endpoint section, the capacity of TS or CQ prophylaxis to prevent WHO stage 2 illnesses will be evaluated to permit assessment of a broader range of disease and illness prevention.

#### The occurrence of all suspected and also laboratory-confirmed infection with bacteria or malaria

TS prophylaxis is expected to prevent both malaria and bacterial infections. Other studies document TS prophylactic efficacy in areas of underlying high rates of bacterial and malarial resistance [3, 4, 19], and the current study will evaluate the effect of TS in this population. Due to the return of CQ-sensitive *Plasmodium falciparum* to Malawi, CQ is expected to be highly efficacious at preventing malaria, but not opportunistic infections.

#### The occurrence of grade 3 or above adverse events and rate of discontinuation of TS or CQ prophylaxis

As a relatively long-term prophylactic regimen, both daily TS and weekly CQ may cause adverse effects that should be accounted for in the risk-benefit analysis of prophylaxis. Evaluation of the incidence of severe and life-threatening events and deaths in each study arm allows for comparison of a baseline event rate to events in the TS and CQ prophylaxis groups. The incidence of events that lead to discontinuation of prophylaxis is also important when policymakers evaluate potential first-line prophylactic regimens and the need for second-line therapies.

### Exploratory endpoints

#### The occurrence of all bacterial infections with antibiotic-resistant organisms

TS prophylaxis has shown benefit in areas where TS resistance is documented. To confirm this finding, we aim to document the efficacy of TS in preventing TS-resistant bacterial infections in our study setting.

#### Clinical and parasitological response to antimalarial therapy in cases of uncomplicated malaria

ART may change the pharmacokinetic profile of artemisinin combination therapies (ACT) used for first-line treatment of uncomplicated malaria in Malawi. Documenting the clinical and parasitological response to these antimalarials serves to evaluate their effectiveness. Reduced efficacy of ACT would signal the need for alternate antimalarial therapies in this population.

## Methods/design

**Fig. 1:**
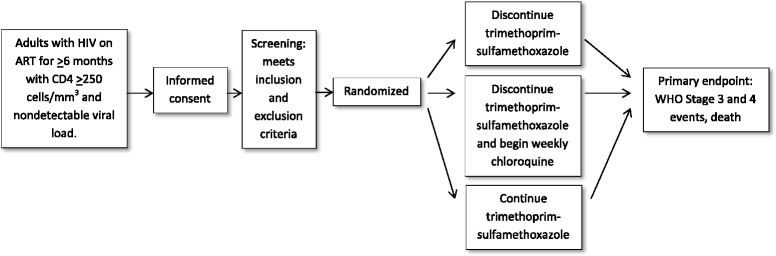
Trial flow diagram

This randomized, controlled, open-label, phase III trial will compare standard of care TS prophylaxis and CQ prophylaxis to no prophylaxis. Adults who have been receiving ART for at least 6 months with good clinical response, and who fulfill the eligibility criteria, will be randomized in a 1:1:1 ratio to one of three arms: (1) to continue standard of care TS prophylaxis, (2) to discontinue standard of care TS prophylaxis and begin weekly CQ prophylaxis, or (3) to discontinue standard of care TS prophylaxis. Participants will be asked to return to the research clinic every 4 weeks for the first 24 weeks then every 12 weeks thereafter, and any time they are ill. Participation will last for 32–66 months. Participants who develop a WHO clinical stage 3 or 4 illness, experience a sustained decline in their CD4 count below 200 cells/mm^3^, or who experience ART failure will be placed on standard of care TS prophylaxis (Fig. 1)

.

The study population is comprised of 1400–1500 Malawian adults living with HIV in or near Blantyre or Zomba, Malawi. Participants must be taking ART for at least 6 months with good clinical response, and have an undetectable HIV viral load and a CD4 count of 250/mm^3^ or more. Major exclusion criteria include severe acute illness, chronic treatment or secondary prophylaxis with any drug with antimalarial or antibacterial activity, a history of hypersensitivity to antifolate drugs or CQ, anemia, thrombocytopenia, neutropenia, liver or kidney failure, and pregnancy. Ethical and regulatory approvals were obtained from the University of Malawi College of Medicine Research Ethics Committee; the Malawi Pharmacy, Medicines and Poisons Board; and the University of Maryland Baltimore Institutional Review Board.

### TS prophylaxis dose

Trimethoprim-sulfamethoxazole (TS) will be provided in tablet form containing 80 mg trimethoprim and 400 mg sulfamethoxazole or 160 mg trimethoprim and 800 mg sulfamethoxazole, manufactured by pharmaceutical companies approved by the US Food and Drug Administration (FDA) or PEPFAR for TS manufacturing. The dose for prophylaxis is either two tablets (each containing 80 mg trimethoprim and 400 mg sulfamethoxazole) or one tablet (each containing 160 mg trimethoprim and 800 mg sulfamethoxazole) to be taken every day by mouth.

### CQ prophylaxis dose

Chloroquine (CQ) will be provided in 500-mg tablet form containing 300 mg chloroquine base, or in 200–250-mg tablet form containing 155 mg chloroquine base, manufactured by pharmaceutical companies approved by the US FDA, Global Fund, or PEPFAR for CQ manufacturing. The dose for prophylaxis is one or two tablets to be taken every 7 days by mouth, for a total weekly dose of 300–310 mg chloroquine base per week.

### Study population

HIV-infected adults enrolled in ART clinics may enroll in the study, provided that they have been taking ART for at least 6 months, complete the informed consent process and fulfill all the inclusion and exclusion criteria.

### Study location

The study recruits participants from Blantyre and Zomba, Malawi.

### Inclusion criteria

- Age 18 years or older
- Documented HIV-1 infection
- Initiation of ART through a government-sponsored ART program at least 6 months previous
- Undetectable HIV viral load (less than 400 copies/mL)
- CD4 count 250/mm^3^ or more
- TS prophylaxis prescribed for at least the previous 2 months
- Intention to remain in the study area until the end of the study period
- Informed consent from participant
- Female study volunteers of reproductive potential must have a negative urine pregnancy test performed within 20 days before randomization
- Female study volunteers of reproductive potential who participate in sexual activity that could lead to pregnancy must use contraception (male or female condoms, diaphragm or cervical cap with spermicide, intrauterine device, or hormone-based contraceptive) while receiving their assigned study drug and for 1 month after stopping the medications

### Exclusion criteria

- Severe acute illness (defined as requiring hospitalization at the time of screening or other conditions such as laboratory abnormalities as determined by the investigators)
- Chronic treatment (requiring therapy for more than 14 days) or secondary prophylaxis (for toxoplasmosis, pneumocystis pneumonia, or tuberculosis for example) with any drug with antimalarial or antibacterial activity
- History of hypersensitivity to antifolate drugs or CQ
- Laboratory exclusion criteria
  - Hemoglobin below 8.0 gm/dL
  - Platelet count below 50,000/mm^3^
  - Absolute granulocyte count below 500/mm^3^
  - Serum alanine aminotransferase (ALT) concentration above 210 U/L for men, and above 160 U/L for women
  - Serum creatinine concentration above 3.3 mg/dl (291.7 μmol/L) for men, and above 2.7 mg/dl (238.7 μmol/L) for women
- History of visual field or retinal changes
- History of preexisting auditory damage
- History of porphyria
- History of psoriasis
- History of liver disease
- History of seizure disorder
- History of glucose-6-phosphate dehydrogenase (G6PD) deficiency
- History of electrocardiogram (ECG) or cardiac conduction abnormality, or cardiomyopathy
- History of myopathy

### Study procedures (Table 2)

#### Recruitment

Individuals enrolled in ART clinics at Ndirande and Zomba are eligible after 6 months of taking ART. The ART clinic staff will inform patients in the ART program about the possibility of enrolling in this study. No study-related evaluations are undertaken before obtaining informed consent. The study staff then ask the participant about their plans to remain in the area and their willingness to attend the research clinic for all scheduled and unscheduled follow-up. Potentially eligible participants are then requested to read the informed consent in either English or Chichewa. The informed consent is read and explained verbally in Chichewa to those who are unable to read. This verbal explanation is attended by a witness who will also sign the consent form. After reading or listening, the potential participant is asked to sign the informed consent form. For those who cannot write, a thumbprint is placed on the form, with the signature and date of a witness. The document also allows the participant to indicate how to handle specimens that remain after the study procedures have been completed. Any use of study samples that is outside the scope of the objectives of this protocol will be submitted for prior review and approval by the appropriate Institutional Review Board (IRB). After informed consent is obtained, participants are given a signed copy of the informed consent document and assigned a screening identification number.

**Table 2:** Trial flow chart

	Screening	Enrollment	Every 4 weeks for 1st 24 weeks, then every 12 weeks	Additional evaluations every 24 weeks	Final study visit, time of termination	Premature discontinuation of study treatment	Unscheduled Visits
Informed consent	√						
Review of past medical history	√						
Review of current complaints		√	√		√	√	√
Medication history	√		√		√	√	
Bednet use		√	√		√	√	
WHO performance scale		√		√	√	√	
Complete blood count, alanine aminotransferase, creatinine	√			√	√	√	
Urine pregnancy test	√	√^a^	√^a^				
CD4 count	√			√	√	√	
HIV viral load	√			√	√	√	
Filter paper sample		√	√		√	√	
Physical examination	√	√ (limited)	√ (limited)	√	√	√	√
Visual acuity assessment		√		√	√	√	
Provision of medication		√	√				
Pill count and adherence interview			√		√	√	

^a^Urine pregnancy testing will be performed at all study visits only where pregnancy is suspected

#### Screening

After informed consent is obtained, a numbered case record folder is created for potential participants, including demographic information, previous medication exposure, ART history, allergy history and physical examination documentation. The participant is also sent to the laboratory for venipuncture to collect 10–12 mL of blood to test for the following:

- Complete blood count (CBC)
- Alanine aminotransferase (ALT) concentration
- Creatinine concentration
- CD4 count
- HIV viral load

Women of reproductive potential undergo urine pregnancy testing. All potential participants are given a follow-up appointment to find out if the laboratory results are within the eligible range. Screening evaluations to determine eligibility are completed within 20 days of study entry unless otherwise specified.

#### Randomization

Participants meeting all eligibility requirements are randomized to one of the three study arms in a 1:1:1 ratio in real time via an Internet data entry system that meets FDA requirements for electronic records and signatures (21 CFR part 11-compliant). A backup hard copy randomization scheme devised by the study statistician is used in case of Internet system failure. The study statistician has no contact with participants or the study staff who care for the participants. Data entry staff onsite record the study arm assignment for the enrolled participant. The participant then surrenders their current stock of TS and receives a supply of the study drug, if appropriate.

#### Blinding

Treatment arms are not masked as each has a different regimen (TS is once daily while CQ is once weekly), and because of the concern that a complicated placebo regimen may interfere with adherence to ART. This approach facilitates assessment of the effect of each prophylaxis regimen on treatment adherence. Laboratory technicians and the independent committee reviewing potential WHO stage 3 and 4 events are blinded to participant treatment assignment, whereas clinical staff are not.

#### Enrollment

After randomization, baseline measurements of visual acuity using Snellen vision testing, and WHO performance score (Table 3) and bednet use (Table 4) are assessed and recorded. A dried blood spot specimen is obtained. The participant is then escorted to the study pharmacy. For those randomized to receive TS or CQ, a supply of the study drug will be given to each participant with instructions for how to take the medication. Participants will be asked to provide detailed directions to their home and of their contact cell phone number, if available, to facilitate active follow-up.

**Table 3:** World Health Organization performance score

World Health Organization performance score	Definition
1	Asymptomatic, normal activity
2	Symptomatic, normal activity
3	Bedridden <50 % of the day during the last month
4	Bedridden ≥50 % of the day during the last month

**Table 4:** Bednet use data collection

Uses bednet	☐ Yes	☐ No
If yes:		
Used most nights in last 4 weeks?	☐ Yes	☐ No
Used last night?	☐ Yes	☐ No

#### Follow-up

Follow-up visits may be one of three types: routine visit, unscheduled visit, or antimalarial efficacy follow-up.

Routine visits occur every 4–12 weeks (every 4 weeks for the first 24 weeks, then every 12 weeks thereafter). Initially, to ensure participant compliance with the study schedule and study drug regimen (if assigned to TS or CQ), participants will be followed every 4 weeks for the first 24 weeks. Thereafter, participants will be followed every 12 weeks, as is the standard of care for ART clinics in Malawi. The encounter will include an interval history, regimen adherence questionnaire (Table 5) and pill counts, questions about off-study drug use and bednet use (Table 4) and a physical examination. In addition, study clinicians will record and/or update any AEs. Dried blood spot samples are obtained, and women of reproductive potential whose last menstrual period was more than 4 weeks prior to this, or whose clinical evaluation suggests pregnancy, will undergo urine pregnancy testing. At routine visits every 24 weeks and the final study visit, approximately 10–12 mL of blood are drawn for determination of CD4 count, HIV viral load, CBC, and ALT and creatinine concentrations; visual acuity is assessed and the WHO performance score (Table 3) is recorded. When participants are followed every 12 weeks, the study team contacts them via cell phone (if number is provided) every 4–6 weeks to ask about their health and to request that they come to the clinic for any current health problems.

**Table 5:** Regimen adherence questionnaire

1. How often do you feel that you have difficulty taking your HIV medications on time? By “on time” we mean no more than 2 hours before or 2 hours after the time your doctor told you to take it. ☐ All of the time ☐ Most of the time ☐ Rarely ☐ Never
2. On average, how many days *per week* would you say that you missed at least one dose of your HIV medications? ☐ Every day ☐ 4 to 6 days/week ☐ 2 or 3 days/week ☐ Once a week ☐ Less than once a week ☐ Never
3. When was the last time you missed at least one dose of your HIV medications? ☐ Within the past week ☐ 1 to 2 weeks ago ☐ 3 to 4 weeks ago ☐ Between 1 and 3 months ago ☐ More than 3 months ago ☐ Never

During unscheduled visits, participants are evaluated according to standard procedures for the evaluation and treatment of illness as defined by the Ministry of Health. Illnesses and other medical issues are recorded as AEs in the participant records, and seriousness, severity, relationship to study product and expectedness are recorded (Table 6). If an unexpected adverse drug experience is observed that is definitely or probably related to TS or CQ, it will be reported to the US FDA using the MedWatch safety information and adverse reporting system via the online system at www.fda.gov/MedWatch/report.htm. Diagnoses of illnesses experienced by participants will be documented based on updated criteria for diagnoses established by the ACTG group (http://www.hptn.org/web%20documents/HPTN052/Appendix60V1.123Feb2007.pdf) when feasible in this setting. If bacteremia or meningitis is suspected, a specimen is collected for culture and isolated pathogens undergo drug susceptibility testing. Any signs or symptoms of malaria will prompt the collection of a malaria smear. Participants who are diagnosed with malaria receive therapy according to Malawi national policy.

**Table 6:** Classification of adverse events

Seriousness	A serious adverse event includes any untoward medical occurrence that at any dose: • Results in death, • Is life-threatening, • Requires inpatient hospitalization or prolongation of existing hospitalization • Results in persistent or significant disability/incapacity, or • Is a congenital anomaly/birth defect Important medical events that may not be immediately life-threatening or result in death or hospitalization but may jeopardize the patient or may require intervention to prevent one of the outcomes listed in the definition above may also be considered serious		
Severity	Grade 1	Mild	Symptoms causing no or minimal interference with usual social and functional activities
	Grade 2	Moderate	Symptoms causing greater than minimal interference with usual social and functional activities
	Grade 3	Severe	Symptoms causing inability to perform usual social and functional activities
	Grade 4	Potentially life- threatening	Symptoms causing inability to perform basic self-care functions *or* medical or operative intervention indicated to prevent permanent impairment, persistent disability
	Grade 5	Death	
Relationship to study products	Definitely related	The adverse event and administration of the study agent are related in time, and a direct association can be demonstrated	
	Probably related	The adverse event and administration of the study agent are reasonably associated in time, and the adverse event is more likely explained by the study agent than other causes	
	Possibly related	The adverse event and administration of the study agent are reasonably related in time, and the adverse event can be explained equally well by causes other than the study agent	
	Probably not related	A potential relationship between the study agent and the adverse event could exist (i.e. the possibility cannot be excluded), but the adverse event is most likely explained by causes other than the study agent	
	Not related	The adverse event is clearly explained by another cause not related to the study agent	
	Pending	Pending may be used as a temporary relationship assessment only for death and only if data necessary to determine relationship to the study agent are being collected. The site is required to submit a final assessment within three business days after reporting the death. If no final assessment is made within three business days after the date of submission, the event will be assessed as possibly related to the study agent. Any additional information received at a later time, including an autopsy report, should be submitted as a Follow-up Report	
Expectedness	Expected	Expected refers to the perspective of events previously observed, not on the basis of what might be anticipated from the pharmacological properties of the study agent	
		Study drug	Expected adverse events
		Trimethoprim-sulfamethoxazole	Rash, urticaria, loss of appetite, nausea, vomiting, agranulocytosis, aplastic anemia, disease of the hematopoietic system, fulminant hepatic necrosis, severe allergic reaction, Stevens-Johnson syndrome, and toxic epidermal necrolysis
		Chloroquine	Headache, malaise, dizziness, blurred vision, difficulty focusing, muscle weakness, electrocardiogram changes, gastrointestinal upset, mouth ulcers, diarrhea, vomiting, nonurticarial pruritis, leukopenia, methemoglobinemia, and retinopathy
	Unexpected	Unexpected refers to events whose nature or severity (intensity) is not consistent with those included in the package insert/summary.	

Participants who are diagnosed with uncomplicated clinical malaria are enrolled in a 28-day antimalarial efficacy study with follow-up on days 1, 2, 3, 7, 14, 21 and 28. On these days, finger-prick specimens for malaria smears and dried blood spot collection are obtained (Table 7). Standard definitions of adequate parasitological and clinical response to antimalarial therapy will be used based on current WHO guidelines (http://whqlibdoc.who.int/publications/2009/9789241597531_eng.pdf). Participants who have clinical treatment failure will be administered rescue therapy according to Malawi national policy. Cases of severe malaria are referred to the hospital.

**Table 7:** Antimalarial efficacy flow chart

Days after malaria diagnosis →	0	1	2	3	7	14	21	28	Unscheduled visit (before d28)
Procedure ↓									
Clinical Assessment	C	C	C	C	C	C	C	C	C
Temperature	C	C	C	C	C	C	C	C	C
Questioning about antimalarial drug use	C	C	C	C	C	C	C	C	C
Finger stick blood sample for malaria smear	C	R	R	R	R	R	R	R	C
Finger stick blood sample for filter paper sample for PCR analysis	R	R	R	R	R	R	R	R	R
Administration of antimalarial drug (per Malawi Ministry of Health Protocol)	C	C	C						

*C*= procedures that are part of clinical care

R= research procedures

### Antiretroviral therapy

Outpatient clinical care of study participants, including ART management, is assumed by the study team at enrollment. The study pharmacist dispenses ART from the government supply to participants at scheduled follow-up visits. The study team provides adherence counseling to participants when missed doses are noted or when ART failure is suspected. Physicians specializing in HIV care hold weekly hours at the research clinic to manage complicated ART questions and HIV care.

### Concomitant medications

At each visit, information on other medications and supplements will be documented in the participant case record forms, including indication, dose, frequency, and start and stop dates.

### Toxicity management

For TS, related AEs are managed according to treatment guidelines based on the WHO grading scale for toxicity in adults and adolescents (http://www.who.int/hiv/pub/guidelines/ctxguidelines.pdf). Other AEs associated with the study drug will be managed by the study team.

For CQ, participants who experience a worsening in visual acuity (defined as a change in two levels or more using Snellen testing) or other visual complaints, muscle weakness, or hearing defects while taking CQ will discontinue CQ prophylaxis and will be referred to the ophthalmology or appropriate clinic for further evaluation and treatment. They will be placed on TS prophylaxis after CQ is discontinued.

### Discontinuation of study treatment assignment

If a participant is diagnosed with a WHO clinical stage 3 or 4 illness during the course of the study, or if their CD4 count is confirmed at below 200 cells/mm^3^, or they experience ART failure, they are given daily TS prophylaxis and followed according to the study protocol. If they had been assigned to CQ prophylaxis, the CQ will be stopped. They will not be withdrawn from the study for this reason.

If a participant becomes pregnant during the study, she will be given TS prophylaxis and remain in the study. If she was previously randomized to daily TS, she will continue taking daily TS. If she was previously randomized to take weekly CQ, she will discontinue weekly CQ. If she was previously randomized to no prophylaxis, she will begin taking daily TS. Pregnancy outcome information will be recorded on a pregnancy outcome case report form (CRF). When the participant is no longer pregnant, she may recommence the treatment to which she was originally assigned, if this is agreed to by the participant and the study team.

### Criteria for permanent treatment discontinuation

- Requires chronic treatment with an antimalarial drug
- Study drug-related toxicity such that reintroduction or desensitization cannot be considered per WHO guidelines
- Request by the subject to terminate treatment
- Clinical reasons believed to be life-threatening by the investigators, such as severe allergic reaction

### Criteria for premature study discontinuation

- Request by the subject to withdraw
- Opinion of the investigators is that the study is no longer in the best interest of the subject
- Volunteer is judged by the site investigator to be at significant risk of failing to comply with the provisions of the protocol, so as to cause harm to self or seriously interfere with the validity of the study results
- Discontinuation at the discretion of the study team, ethics committee, or the study sponsor

### Final study visit

At the final study visit, the WHO performance score will be measured and approximately 10–12 mL of blood will be drawn for CD4 count, HIV viral load, CBC, and ALT and creatinine concentrations. Participants will discontinue their study treatment assignments and will receive TS prophylaxis according to Malawi national policy. Management of their HIV care will be transferred to the government-sponsored ART clinic.

### Definition and assessment of adverse events

#### Definition of adverse event

An adverse event (AE) is any untoward medical occurrence in a patient or clinical investigation participant who is administered a study product/intervention(s). An AE does not necessarily have a causal relationship with study treatment. An AE can, therefore, be any unfavorable and unintended sign (including an abnormal laboratory finding), symptom, or disease temporally associated with the use of a medicinal (investigational) study product/intervention(s), whether or not related to the medicinal (investigational) study product/intervention(s).

An unanticipated problem (UP) is defined as an event (including onsite and offsite AE reports, injuries, side effects, breaches of confidentiality, deaths or other problems) that occurs any time during or after the research study, which, in the opinion of the principal investigator (PI):

- Involves harm to one or more participants or others, or places one or more participant or others at increased risk of harm *and*
- Is unexpected *and*
- Is related to the research procedures

The occurrence of an AE may come to the attention of study personnel during study visits and interviews or by a study participant presenting for medical care, or upon review by a study monitor. Any medical condition that is present at the time the participant is enrolled will be considered a baseline condition, and not reported as an AE. However, if it deteriorates at any time during the study it will be recorded as an AE.

AEs will be captured on the appropriate case report forms. AEs will be recorded in study source documents, and an assessment of whether they are associated with the study drug, the ART regimen, an intercurrent illness or another cause will be made. Information to be collected includes event description, date of onset, clinician’s assessment of seriousness and severity, relationship to study product, expectedness and time of resolution/stabilization of the event. AEs will be followed to adequate resolution or stabilization. If the AE has not stabilized or resolved at the end of the study period, it will be followed for a maximum of 1 year after study completion.

#### Seriousness of event

AEs will be assessed by study clinicians to determine the seriousness of the outcome of the event. The April 1996 International Conference on Harmonization (ICH) guidance, “Good Clinical Practice: Consolidated Guidance,” (ICH E6) defined a serious adverse event (SAE) as “any untoward medical occurrence that at any dose:

- Results in death
- Is life-threatening
- Requires inpatient hospitalization or prolongation of existing hospitalization
- Results in persistent or significant disability/incapacity, or
- Is a congenital anomaly/birth defect.”

Important medical events that may not be immediately life-threatening or result in death or hospitalization but may jeopardize the patient or may require intervention to prevent one of the outcomes listed in the definition above may also be considered to be serious. (October 1994 ICH guidance (E2A), “Clinical Safety Data Management: Definitions and Standards for Expedited Reporting.”)

#### Severity of event

AEs will be assessed by the investigator using a protocol-defined grading system (Table 8) and the Division of AIDS (DAIDS) Adult Toxicity Tables. (http://rsc.tech-res.com/safetyandpharmacovigilance/) or locally derived normal ranges (for creatinine and ALT concentrations). For events not included in the DAIDS Adult Toxicity Tables or protocol-defined grading system, the following guidelines will be used to quantify intensity for clinical AEs:

- Grade 1 mild: symptoms causing no or minimal interference with usual social and functional activities
- Grade 2 moderate: symptoms causing greater than minimal interference with usual social and functional activities
- Grade 3 Severe: symptoms causing inability to perform usual social and functional activities
- Grade 4 Potentially life-threatening: symptoms causing inability to perform basic self-care functions *or* medical or operative intervention indicated to prevent permanent impairment, persistent disability
- Grade 5 Death

**Table 8:** Severity of grading and use of normal and abnormal laboratory values

	Grade 1	Grade 2	Grade 3	Grade 4
Hemoglobin	8.5–10.0 g/dL	7.5–8.4 g/dL	6.5–7.4 g/dL	<6.5 g/dL
Absolute neutrophil count	1000–1300/mm^3^	750–999/mm^3^	500–749/mm^3^	<500/mm^3^
Platelets	100,000–124,999/mm^3^	50,000–99,999/mm^3^	25,000–49,999/mm^3^	<25,000/mm^3^
Creatinine	1.1–1.3 × ULN	1.4–1.8 × ULN	1.9–3.4 × ULN	≥3.5 × ULN
Alanine aminotransferase	1.25–2.5 × ULN	2.6–5.0 × ULN	5.1–10.0 × ULN	>10.0 × ULN

*ULN* upper limit of normal

When intensity changes occur more frequently than once a day, the maximum severity for the event should be listed.

#### Relationship to study products

AEs will have their relationship to study product assessed using the following terms:

- *Definitely related*. The AE and administration of the study agent are related in time, and a direct association can be demonstrated
- *Probably related*. The AE and administration of the study agent are reasonably associated in time, and the AE is more likely explained by study agent than other causes
- *Possibly related*. The AE and administration of the study agent are reasonably related in time, and the AE can be explained equally well by causes other than the study agent
- *Probably not related*. A potential relationship between the study agent and the AE could exist (i.e., the possibility cannot be excluded), but the AE event is most likely explained by causes other than the study agent
- *Not related*. The AE is clearly explained by another cause not related to the study agent
- *Pending*. Pending may be used as a temporary relationship assessment only for death and only if data necessary to determine the relationship to the study agent are being collected. The site is required to submit a final assessment within three business days after reporting the death. If no final assessment is made within three business days after the date of submission, the event will be assessed as possibly related to the study agent. Any additional information received at a later time, including an autopsy report, should be submitted as a Follow-up Report

A suspected adverse drug reaction (SADR) is an AE that could potentially have a causal relationship to the study agent (definitely, probably, possibly, probably not related, or for deaths, pending).

#### Expectedness (expected versus unexpected)

Expected refers to the perspective of events previously observed, not on the basis of what might be anticipated from the pharmacological properties of the study agent (ICH E2A). Expected AEs related to study drugs are found in Table 6.

Unexpected refers to events whose nature or severity (intensity) is not consistent with those included in the package insert/summary (ICH E2A). If an unexpected adverse drug experience is observed that is definitely or probably related to TS or CQ, it will be reported to the US FDA using the MedWatch safety information and adverse reporting system via the online system at www.fda.gov/MedWatch/report.htm. An unexpected adverse drug experience is defined as any adverse drug experience that is not listed in the current labeling for the drug product. This includes events that may be symptomatically and pathophysiologically related to an event listed in the labeling but differ from the event because of greater severity or specificity. “Unexpected,” as used in this definition, refers to an adverse drug experience that has not been previously observed (i.e., included in the labeling) rather than from the perspective of such experience not being anticipated from the pharmacological properties of the pharmaceutical product.

### Adverse event reporting

Only serious, unexpected, related AEs and all deaths will be reported in an expedited manner.

Regulatory requirements for reporting to the University of Malawi, the University of Maryland and the DAIDS clinical representative will be observed. Reporting procedures will follow ICH 4.11, 5.17 and Clinical Safety Data Management: Definitions and Standards for Expedited Reporting.

### Protocol deviations

Protocol deviations are events that contradict or omit protocol instructions. Violations that compromise patient safety or the integrity of trial data will be recorded and reported to the sponsor and to the responsible regulatory authorities, including ethics committees, as required by the regulations of each group.

### Statistics

#### Sample size and power calculations

The study will randomize approximately 467 participants to each of the three study arms during a 36-month enrollment period and will follow participants for 32–66 months. We estimate that loss to follow-up will amount to approximately 15 % of the potential follow-up time, so that the follow-up time available for analysis will average about 41.6 months per study participant, for a total of approximately 1619 person-years of follow-up in each study arm.

For estimating the power of the study, the statistical null hypothesis is that the effect of TS prophylaxis in preventing the first occurrence of a severe event, relative to no prophylaxis, is at least a 35 % reduction in the hazard rate for first occurrences over the study period (i.e., that the hazard ratio, HR, is ≤0.65). Our assumption (alternative hypothesis) for power calculation is that there is no preventive effect of TS, i.e., that HR = 1. The comparison will be based on the upper limit of a two-sided 95 % confidence interval (CI) for the TS effect. An upper limit of less than 35 % – i.e., a reduction significantly less than 35 % at the one-sided 2.5 % significance level – will be considered sufficient evidence for discontinuing TS prophylaxis in this population.

Assuming that the number of severe events follows a Poisson distribution, the mean of the distribution for a participant with average follow-up time (41.6 months) will be approximately 0.236 events, and the probability that the participant will experience at least one severe event will be about 0.210. Assuming that this incidence rate applies to participants taking TS prophylaxis and those without prophylaxis (i.e., no effect of TS prophylaxis), we then expect for the comparison about 196 study participants in both study arms combined to experience at least one severe event, which gives us an expectation of about 85 % power that the upper limit of the 95 % CI for the effect of TS will be less than 35 % [20]. The same consideration applies to the comparison of discontinuation of prophylaxis and CQ prophylaxis.

### Analysis

#### Analysis of the primary endpoint

The primary analysis will compare continuation of TS and discontinuation of TS. It will consist of a 95 % CI for the effect of TS, relative to no prophylaxis, in preventing the occurrence of a severe event, based on proportional hazards (Cox) regression modeling. The TS effect is defined as 1 − HR, where HR is the hazard ratio from the Cox regression model. The analysis will be done according to the intention-to-treat principle; that is, each individual’s data will be analyzed according to the study arm to which the individual was randomized, regardless of the treatment and amount of treatment actually received. Study participants who develop a WHO clinical stage 3 or 4 illness, experience a sustained decline in their CD4 count below 200 cells/mm^3^, or who experience ART failure will be given TS prophylaxis and will continue to be followed in the study. The analytical method for comparing CQ prophylaxis and no prophylaxis (i.e., discontinuation of TS) will be the same as for comparing continuation and discontinuation of TS.

In addition, one or more protocol analyses may be done if there is substantial lack of adherence or administration of a treatment other than the one to which an individual was randomized. This consideration is particularly important since we will be performing noninferiority analysis of discontinuation of TS prophylaxis.

Secondary analyses will compare TS prophylaxis to no prophylaxis, and CQ prophylaxis to no prophylaxis, with respect to the total number of severe events experienced (i.e., including multiple events in individual study participants), using Poisson regression modeling. TS and CQ prophylaxis will also be compared to each other with respect to the first occurrence and all occurrences of severe events. We do not plan to adjust *p* values or CIs for multiple comparisons.

#### Analysis of loss of viral suppression

Besides severe events, we are interested in assessing the value of TS and CQ prophylaxis in preventing loss of viral suppression. As for the first occurrence of a severe event, separate comparisons of (1) TS continuation and discontinuation, (2) TS continuation and CQ prophylaxis, and (3) CQ prophylaxis and TS discontinuation will be done using Cox regression modeling to estimate two-sided 95 % CIs for treatment effects, expressed as HRs. Since severe events are expected to occur mainly in participants who do not maintain their viral suppression (i.e., who develop a detectable viral load), we define minimally acceptable prophylaxis, as for the occurrence of a severe event, as at least a 35 % effect in preventing loss of viral suppression. Then the same numbers of losses of viral suppression are needed for 80 % power to obtain a CI for effect of prophylaxis with upper bound less than 35 %, assuming there is no effect, as for the first occurrence of a severe event. We expect the probability of maintaining viral suppression to be approximately 75 % for participants taking TS prophylaxis who have the average amount of follow-up (49 months) [21]. Then about 75 participants in each of the study arms will be expected to lose their viral suppression during the study. The power to obtain an upper confidence limit less than 35 % for effect of prophylaxis is then about 75 % for each of the comparisons.

Intention-to-treat analysis will be used for all primary comparisons. This analysis will include all participants who were randomized, regardless of attendance at follow-up visits and the duration of study drug administration. For participants who are no longer being actively followed at the end of the study, every effort will be made to obtain their vital status. Per-protocol analysis will be performed as a secondary analysis for those who complied with follow-up visits at least every 8 weeks and for study and ART medication. Analyses will be done in which data on study volunteers who switch from one study intervention to another are censored at the time that they develop an indication for TS prophylaxis, regardless of treatment assignment.

### Data safety and monitoring

In addition to the regular safety monitoring conducted by the investigators, this trial will be reviewed at least annually by the US National Institutes of Health (NIH) NIAID Data and Safety Monitoring Board (DSMB). An independent biostatistician and/or database management group will prepare the DSMB report for the scheduled DSMB meetings. Follow-up intervals of reporting will be at the discretion of the DSMB based on trends in the data, but these are planned to occur approximately every 6 months for safety and once per year for efficacy. The NIH NIAID DSMB can meet more frequently as needed. The DSMB will be independent from the investigators and will have full unblinded access to all accumulating data. The biostatistician and/or database management group will prepare reports that include:

- Study accrual by month and by study site
- Eligibility violations
- Baseline characteristics
- Protocol adherence report
- Data completeness report
- Periodic summary adverse event report
- Primary and secondary endpoint summaries, overall and for key subgroups

The initial DSMB review is planned specifically to assess safety data to assure that there are not dramatic differences in survival between treatment groups, and to determine the appropriate frequency of DSMB reporting. If the trial executive committee or DAIDS Clinical Representative has efficacy or safety concerns, an unscheduled DSMB review will be requested.

Site monitors under contract to the National Institute of Allergy and Infectious Diseases (NIAID) DAIDS will visit participating clinical research sites to review individual subjects’ records, including consent forms, CRFs, supporting data, laboratory specimen records, and medical records (physicians’ progress notes, nurses’ notes, individuals’ hospital charts), in order to ensure protection of study subjects, compliance with the protocol, and accuracy and completeness of records. The monitors also will inspect sites’ regulatory files to ensure that regulatory requirements are being followed and sites’ pharmacies to review product storage and management.

The DSMB will review safety and enrollment data approximately every 6 months after the first study participant is enrolled and at other intervals if deemed necessary by the sponsor or study investigators, with input from the DSMB. Datasets for these analyses will be created in a joint effort between the data management team and the independent statistician. The major purpose of these analyses will be to review study progress and safety data. Safety outcome measures to be monitored include SAEs that are deemed probably or definitely associated with the study interventions and the total number of SAEs in each treatment group. The number of primary endpoint events (deaths, WHO stage 3 and 4 events) as well as the number of AEs of grade 3 or more and the rates of discontinuation of TS or CQ prophylaxis, and of loss of virologic control will be included in the safety review. Based on their assessment of study enrollment, the safety and tolerability of the prophylaxis, and any new research evidence from similar clinical trials, the DSMB will make a recommendation that the study either continue with no modification; continue with modification (including the possibility of terminating one of the prophylaxis arms); or be discontinued, either permanently or temporarily pending further review.

## Ethical considerations

### Human subjects’ protections

The investigator will ensure that this study is conducted in full conformity with the principles set forth in The Belmont Report: Ethical Principles and Guidelines for the Protection of Human Subjects of Research of the US National Commission for the Protection of Human Subjects of Biomedical and Behavioral Research (18 April 1979) and codified in 45 CFR Part 46 and/or ICH E6; 62 Federal Regulations 25691 (1997). Key study personnel will maintain certification in human subjects protection and good clinical practice (GCP).

### Ethical review

This protocol, the informed consent document and any subsequent modifications were reviewed and approved by the Institutional Review Board or Ethics Committee responsible for oversight of the study, including the College of Medicine of the University of Malawi and the University of Maryland. The University of Malawi College of Medicine Research Ethics Committee (COMREC) will be considered to be the local IRB and the University of Maryland IRB will be considered to be a remote IRB (Michigan State University IRB will defer to University of Maryland IRB) for the purpose of reporting SAEs [22].

### Informed consent

Informed consent is a process that is initiated prior to the individual’s agreeing to participate in the study and continuing throughout the individual’s study participation. Extensive discussion of risks and possible benefits of this therapy will be provided to the subjects and their families. Consent forms describing in detail the study interventions/products, study procedures, and risks are given to the subject and written documentation of informed consent is required prior to starting intervention/administering study product. Consent forms will be IRB-approved and the subject will be asked to read and review the document, or else verbally explained to the subject (verified by a witness). Upon reviewing the document, the investigator will explain the research study to the subject and answer any questions that may arise. The subjects will sign the informed consent document prior to any procedures being carried out specifically for the study. The subjects should have the opportunity to discuss the study with their surrogates and adequately consider it prior to agreeing to participate. Subjects may withdraw consent at any time throughout the course of the trial. A copy of the informed consent document will be given to subjects for their records. The rights and welfare of study subjects will be protected by emphasizing to them that the quality of their medical care will not be adversely affected if they decline to participate in this study. During the informed consent process, participants agree that if their individual data are presented in manuscript form or in scientific meetings, they will be identified by study number and not by name to protect participant confidentiality.

### Potential risks

Potential risks anticipated for the study participants include study drug-specific effects, risks associated with blood drawing, potential increased risk of opportunistic infections, and potential loss of confidentiality regarding HIV status.

The drugs used in this trial are not investigational new drugs. They have both been widely used for many decades throughout the world and particularly in Africa, at the doses and for the indications they will be used for in this trial. Their safety profiles are favorable and well known. However, as with any drug, risks do exist. Though CQ is considered safe, adverse reactions to this drug may occur. Commonly reported symptoms include headache, malaise, dizziness, blurred vision, difficulty focusing, muscle weakness and mild gastrointestinal upset. Nonurticarial pruritis, without rash, is a problem that is more common among dark-skinned patients. The symptom usually begins within the first day after the initial dose and may last for up to 7 days. Severe adverse reactions are extremely rare [23]. CQ is well known to be safe in pregnancy and is routinely recommended for both malaria treatment and malaria prophylaxis in pregnant women.

Common adverse reactions associated with TS use include rash, urticaria, loss of appetite, nausea and vomiting. Rare, serious side effects include agranulocytosis, aplastic anemia, disease of the hematopoietic system, fulminant hepatic necrosis, severe allergic reaction, Stevens-Johnson syndrome, and toxic epidermal necrolysis [24]. TS is considered to be relatively contraindicated in pregnancy (class C) because of theoretical risks of neural tube defects in the first trimester and kernicterus in the third trimester. However, both the WHO [13] and the Malawi Ministry of Health presently recommend TS prophylaxis for people living with HIV (PLHIV), including in pregnant women.

Finger pricks and venipuncture are associated with small risks of bleeding, hematoma and infection. To minimize this risk, the skin will be cleaned with alcohol prior to puncture and sterile, unused needles and lancets will always be used and pressure will be held at the puncture site after removal of the needle or lancet. Although the quantity of blood drawn would not lead to any ill effects on the participants’ health, some adults feel faint following phlebotomy. The risks will be minimized by having trained technicians perform the procedure. Clinicians will be available to evaluate the participant if there is any untoward effect.

Study subjects who are assigned to the CQ or no treatment arms may be at increased risk for opportunistic infections. However, the fact that immune reconstitution will have already taken place in these individuals reduces this theoretical risk substantially [25, 26]. Additionally, the risk of TS prophylaxis-associated adverse reactions must be weighed against the unknown, potential benefit of TS prophylaxis in this population. The potential risk of increased infection in those not taking TS will be minimized by close follow-up and monitoring, which will lead to prompt diagnosis and treatment of bacterial infections and malaria should they occur.

As with any study involving HIV-positive individuals, there is a risk of loss of confidentiality regarding HIV status. Our study team is well known in the community for conducting studies of malaria, so clinic attendance and home visits will not necessarily identify participants’ HIV status. Efforts to reduce this risk will be a priority. Patients’ medical records will be kept in a locked cabinet in a locked room. The study documentation, data and all other information generated will be kept in strict confidence. No personal information will be released to any unauthorized third party without the consent of the participant. Participant specimens and case report forms (CRFs) will be identified by a study code with the master key will be kept in a separate, locked cabinet.

### Benefits

Study participants will receive a higher standard of medical care than is typically available in Malawi. Close follow-up is likely to identify HIV-related illnesses sooner than they would otherwise be detected. We will pursue complete diagnostic evaluation of illness episodes and maintain a supply of medication to treat common diseases.

### Alternatives to study participation

Participants may withdraw from the study at any time. Those who withdraw will be referred for treatment at the local government-sponsored ART clinic. The reason for withdrawal will be recorded. Whenever possible, endpoint data will be collected for participants who withdraw or are withdrawn from the study in order to include their data in the intention-to-treat analysis.

### Sample sharing and storage

Samples collected will be maintained after the study is complete if the participant has agreed to this during the informed consent process and indicated permission on the written document. Any analysis of these specimens that is outside the scope of the objectives of this protocol will be submitted for prior review and approval by the appropriate IRBs. Parasites, viruses and their genetic material may be unlinked for further analysis, without any identifying clinical information. Should a participant change their mind at any time and revoke authorization for specimen storage with identifying information, their remaining samples will be unlinked from identifying information prior to analysis or else destroyed at the participant’s request.

## Data

### Data collection and entry

The PI is responsible for ensuring the accuracy, completeness, legibility and timeliness of the data reported. All source documents will be completed in a neat, legible manner to ensure accurate interpretation of data. Records will be kept in locked files.

Copies of the electronic CRF (eCRF) will be provided for use as source documents and maintained for recording data for each subject enrolled in the study. Data reported in the eCRF derived from source documents should be consistent with the source documents, or the discrepancies should be explained.

All source documents and laboratory reports will be reviewed by the study team and data entry staff to ensure that they are accurate and complete. AEs must be graded, assessed for severity and causality, and reviewed by the PI, an investigator, or a clinical coordinator. Data collection is the responsibility of the clinical trial staff at the Blantyre Malaria Project Research Clinic and at Dignitas International under the supervision of a clinical coordinator and the investigators. During the study, the investigator must maintain complete and accurate documentation for the study. The Statistical and Data Coordinating Center for this study will be responsible for the data management, quality review, analysis, and reporting of the study data.

Appropriate medical and research records will be maintained for this trial, in compliance with ICH E6 and any applicable DAIDS policies. Study investigators and the clinical coordinator will have access to all study records. Study nurses and clinicians will have access to the laboratory source documents and CRFs of participants who are being actively enrolled and followed. Laboratory staff will have access to laboratory source documents. Data entry clerks and the data manager will have access to the CRFs. Authorized representatives of DAIDS will be permitted to examine (and when required by applicable law, to copy) clinical records for the purposes of quality assurance reviews, audits and evaluation of study safety and progress. The final trial dataset will be available to study investigators at the end of the study.

### Quality control and quality assurance

The site developed a protocol-specific quality management plan in conjunction with the University of Maryland Center for Vaccine Development Office of Regulatory Affairs and Quality Management. This plan will be in place for quality management, including how the data will be evaluated for compliance with protocol, which documents will be reviewed and methods of training staff. The study will be conducted at the Blantyre Malaria Project Research Clinic at Ndirande Health Centre and at the Tisungane Clinic at Zomba Central Hospital in Zomba. Recruitment activities will occur at outpatient ART clinics at these sites. Laboratory testing will also be performed at certified laboratories for testing according to the protocol analyte list.

Site monitoring will be conducted by a DAIDS contractor to assure protocol compliance, ethical standards, regulatory compliance and data quality.

Following written standard operating procedures (SOPs), the monitors will verify that the clinical trial is being conducted and data are generated, documented (recorded), and reported in compliance with the protocol, GCP and the applicable regulatory requirements. Reports will be submitted to DAIDS on monitoring activities. The investigative team will provide direct access to source data/documents and reports for the purpose of monitoring and auditing by the sponsor and inspection by local and regulatory authorities. The data manager will implement quality control procedures beginning with the data entry system and generate data quality control checks that will be run on the database. Any missing data or data anomalies will be communicated to the site for clarification and resolution.

### Monitoring

Study monitoring will be conducted to ensure participant safety compliance of study conduct with 45 CFR 46, GCP, ICH and DAIDS guidelines. A separate monitoring plan will be developed by DAIDS and implemented by the DAIDS Clinical Site and Study Monitoring Contractor.

The site monitors will visit participating clinical research sites to review individual subject records, including consent forms, CRFs, supporting data, laboratory specimen records, and medical records (physicians’ progress notes, nurses’ notes, individuals’ hospital charts), and to ensure protection of study subjects, compliance with the protocol and accuracy and completeness of records. The monitors also will inspect sites’ regulatory files to ensure that regulatory requirements are being followed and sites’ pharmacies to review product storage and management.

### Plans for dissemination of results

The PI and co-investigators regularly participate in local and national meetings in Malawi to discuss malaria and HIV issues as well as local research meetings at the University of Malawi College of Medicine. The findings will be presented at the annual College of Medicine Research Dissemination Day and at other appropriate conference venues. Study results will also be shared expeditiously with Malawian HIV and malaria control officials and the Malawi Ministry of Health and its local representatives. Results will be submitted for publication to local and international journals in accordance with authorship guidelines established by the International Committee of Medical Journal Editors. The study protocol will be provided in publication supplementary materials.

## Discussion

### Clinical trial coordination

Daily study activities are managed onsite by a study physician, with visits twice weekly by a senior study physician with HIV management expertise. Challenging clinical cases are scheduled on days when senior physicians are onsite. The local study teams participate in weekly teleconferences with international investigators to discuss study progress, challenges and planning. SAEs are identified by the study team and reported to senior physicians and international investigators via email and/or phone to refine diagnostics and management as needed. International investigators make regular visits to the study sites approximately every other month to review progress and assist in study coordination.

### Trial status

The study began recruitment activities at the Ndirande site in November 2012. By mid-February 2015, 900 participants had been recruited. The DSMB evaluated study progress as planned, starting in 2013, and determined that the study was being conducted well and should continue.

During the first year of follow-up in 2013, study investigators noted several pregnancies in study participants who had agreed to practice contraception during the informed consent process. As a result, the study team implemented checks at scheduled follow-up visits to ensure that participants were able to access family planning methods in the community, and to facilitate referral to family planning clinics for contraception. As a result, the incidence of pregnancy in the study population has decreased.

Initially, the trial was designed to recruit 900 participants, with the rationale that the primary endpoint event rate in the entire participant population would be 15 per 100 person-years. As no prospective data were available at the time, this assumption was based on a post-hoc analysis of retrospective data from a similar population. As the study accrued participants, the overall event rate was reviewed regularly due to this uncertainty, and the event rate observed in the study population was 6.8 per 100 person-years as of an interim database lock of July 2014. Sample size determination was revisited at that time, and it was determined that adding approximately 500–600 participants and 2 years of follow-up should provide the expected 350 primary endpoint events needed for primary endpoint analysis. The sponsor agreed to extend and expand the study in early 2015, and a second site, Zomba, was added for recruitment and follow-up in mid-2015. A Standardized Protocol Items: Recommendations for Interventional Trials (SPIRIT) checklist is provided in the supplementary materials as Additional file 1.

## Abbreviations

AE, adverse event; ALT, alanine aminotransferase; ART, antiretroviral therapy; CBC, complete blood count; CI, confidence interval; COMREC, College of Medicine Research Ethics Committee; CQ, chloroquine; CRFs, case report forms; DAIDS, Division of AIDS; DART, Development of Antiretroviral Therapy for Africa; DSMB, Data Safety and Monitoring Board; eCRF, electronic case report form; FDA, Food and Drug Administration; G6PD, glucose-6-phosphate dehydrogenase; ICH, International Conference on Harmonization; IRB, Institutional Review Board; NIAID, National Institute of Allergy and Infectious Diseases; NIH, National Institutes of Health; PCP, *Pneumocystis jirovecii* pneumonia; PML, progressive multifocal leukoencephalopathy; SADR, suspected adverse drug reaction; SAE, severe adverse event; TS, trimethoprim-sulfamethoxazole; WHO, World Health Organization.

## Additional file

Additional file 1: Completed SPIRIT checklist. Standardized Protocol Items: Recommendations for Interventional Trials checklist. (PDF 90 kb)
